# Effect of Photobiomodulation on Atrophic–Erosive Clinical Forms of Oral Lichen Planus: A Systematic Review

**DOI:** 10.3390/dj10120221

**Published:** 2022-11-27

**Authors:** Juan Antonio Ruiz Roca, Pía López Jornet, Francisco José Gómez García, Paula Marcos Aroca

**Affiliations:** Department of Dentistry, University of Murcia, 30100 Murcia, Spain

**Keywords:** oral cancer, oral lichen planus, photobiomodulation, atrophic–erosive lesions

## Abstract

Introduction. Oral lichen planus is a chronic autoimmune inflammatory disease of unknown origin, characterized by various clinical forms of which the atrophic–erosive causes patients the greatest symptomatology. For this reason, there are different treatments that improve the associated signs and symptoms. One of these therapies is photobiomodulation (PBM), which, although new, has a high level of acceptance in dentistry based on evidence. However, there are inconsistent results in its application against lichen planus. The aim of this review was to evaluate the effect of photobiomodulation and its effectiveness as a therapeutic alternative for atrophic–erosive lesions. Material and methods. The databases PubMed, Google Scholar and Cochrane Library were searched to identify studies investigating the photobiomodulation treatment in atrophic–erosive lesions of oral lichen planus. A total of 294 articles were identified, published between 2017 and 2022, and then evaluated; 7 articles that met all the inclusion criteria were included in this study. Results. The type of laser light source used in PBM was the diode laser (four cases), the Nd–YAG laser at the same wavelength of 1064 nm (two cases) and the He–Ne laser (one case). The minimum and maximum wavelengths used were 630 nm and 1064 nm, respectively. Most studies used lesions treated with topical corticosteroids as a control group. The follow-up times of the studies were highly variable. Conclusions. Photobiomodulation is a treatment that competently combats oral lichen planus lesions by improving signs and symptoms, with no known adverse reactions so far, which makes it more beneficial compared to more conventional therapies, such as corticosteroids, for which side effects have been found.

## 1. Introduction

The World Health Organization (WHO) described oral lichen planus (OLP) as “a chronic inflammatory disorder of unknown etiology with characteristic relapses and remissions, displaying white reticular lesions, accompanied or not by atrophic, erosive and ulcerative and/or plaque-type areas. Lesions are frequently bilaterally symmetrical. Desquamative gingivitis may be a feature” [1]. Although its etiology is unknown, it is believed to be autoimmune in nature by observing an imbalance of the immune system mediated by auto-cytotoxic CD8+ T lymphocytes that trigger apoptosis of oral epithelial cells, generating the inflammatory lesion [2]. There are psychological factors such as anxiety, depression and stress that predispose to the manifestation or recurrence of OLP lesions [3,4,5]. The average prevalence of OLP worldwide is 1.27%, with an oscillation between 0.1 and 4% due to geographic and pathogenic factors [6]. It mainly affects women starting from the age of 40, although it has also been seen in children [7]. Histologically, it is characterized by hydropic degeneration or liquefaction of the basal epithelium with the presence of Civatte bodies (apoptotic keratinocytes) and a subepithelial inflammation with a lymphocyte infiltrate, mainly CD8+ T cells in a band shape [8]. These histological findings, according to the WHO, are used for a definitive diagnosis of OLP [8]. OLP lesions are considered an oral potentially malignant disorder (OPMD), defined as “clinical manifestations that carry a risk of developing cancer in the oral cavity, either in a clinically definable precursor lesion or in a clinically normal mucosa” [9]. OLP may have a variable risk of malignant transformation to oral squamous cell carcinoma, from 0.44 to 2.28%, as reported in recent systematic reviews and meta-analyses [10]. The risk factors for malignant transformation are smoking or chewing tobacco [11], presenting the erosive form of OLP, more specifically with ulcerative and/or erosive lesions on the edges [12], advanced age, alcohol consumption, presence of the OLP lesion on the tongue [10], and/or presence of HBV (hepatitis B virus) or HCV (hepatitis C virus) infections [7]. The evolution of OLP is heterogeneous; therefore, patients should be monitored through periodic follow-ups even in the absence of symptoms, in order to identify worrying signs of malignant transformation [13]. White lichen lesions (reticular or plaque-shaped forms) mostly heal spontaneously, while red lesions (atrophic–erosive) need treatment [14]. Among the different treatment modalities is the administration of retinoids, immunosuppressive drugs, antifungal agents, surgery and laser, most of which are aimed at relieving signs and symptoms, as well as at preventing possible recurrences [15]. Even so, it is advisable to have a healthy lifestyle with good oral hygiene, exercise and sufficient rest and relaxation in order to achieve control of OLP outbreaks [16]. Normally, corticosteroid therapy is the first choice; however, side effects such as insomnia, mood swings, fatigue, fluid retention, nausea, dry mouth, sore throat, thinning of the oral mucosa and yeast overgrowth may appear [15]. Regarding the most used topical corticosteroids for the treatment of lichen planus, triamcinolone acetonide formulated at 0.3–0.5% is used, followed by fluocinolone acetonide at 0.05% and finally propionate of clobetasol at a concentration of 0.025–0.05% [14,17]. Another treatment option is photobiomodulation (PBM), in which a laser or a non-ionizing radiation (LED) (at 400–1.100 nm wavelenght) is used to beneficially influence cell metabolism, without harm to the cells or to the basal temperature of the body [18]. Different types of laser light such as ultraviolet, diode and helium–neon are used, as well as different output powers, irradiation times, doses and number of sessions for each OLP lesion [19,20]. Although there is no standardized protocol, Del Vecchio et al. [18], in 2021, defined a dose oscillating between 2 and 3 J/cm^2^ as effective in OLP treatment to obtain the desired biological effects [21]. Diodes are often used in dentistry and serve as a preventive treatment for oral mucositis caused by chemotherapy and radiotherapy applied in cancer treatment [22] and to reduce the symptoms of OPMDs [21], as in the case of OLP. This is due to their beneficial effects at the cellular level, such as on proliferation at lower doses of energy and apoptosis at higher doses of energy [23], and at the systemic level, with an analgesic action. PBM was shown to reduce pain and promote clinical improvement of OLP lesions [24], with a decrease in size and erythema, using wavelengths between 630 and 980 nm with an output power of 20 to 300 mW and an exposure time of 10 s to 15 min [25]. These beneficial effects of PBM on OLP would be explained by its ability to delay cell differentiation, improve healing and re-epithelialization, reduce inflammation through immunomodulation and exert an analgesic effect (through the production of beta-endorphins and enkephalins and the reduction of histamine and bradykinin, in addition to the reduction of the activity of sensitive C fibers) [18]. Although more studies are needed and sometimes there are contradictory results, the latest research suggests that PBM could be just as effective as topical corticosteroids, but without their adverse effects, which makes it a very promising therapy [26]. The aim of this systematic review w2as to provide a synthesis of the scientific evidence of PBM usefulness in oral medicine and its contribution to improving the quality of life of patients. It focuses on–atrophic– erosive lichen planus since this is the OLP form that reduces the most the quality of life of patients and is considered at the greatest risk for malignant transformation. The purpose of this systematic review was to determine the effects of PBM on the atrophic–erosive form of OLP by in relation to the physical parameters of the laser, the stimulation of healing, the improvement of painful symptoms and the anti-inflammatory effects, comparing its effectiveness with other treatments, such as corticosteroids. Our hypothesis was that PBM could be an effective therapeutic alternative to conventional treatments.

## 2. Materials and Methods

In this systematic review, the criteria of the PRISMA [27] declaration were met at all times. In addition, it should be noted that both the research question posed and the search method applied were established following the PICO strategy (Table 1), which is very characteristic for clinical research. “PICO” question: Patients with atrophic–erosive OLP (P = Patient); PBM treatment with laser (I = Intervention); laser off, or drug (C = Comparison); remission of symptoms (O = Outcome) (Table 1). The protocol was registered in the international prospective register of systematic reviews PROSPERO (CRD42019154002).

### 2.1. Selection of the Studies and Eligibility Criteria: Inclusion and Exclusion Criteria

This systematic review was carried out by two researchers (JARR and PMA) who focused their search on articles indexed in the Journal Citation Reports (JCR) in which PBM was used in the treatment of atrophic–erosive OLP. Three databases were analyzed: PubMed (Medline), Cochrane Library and Google Scholar, limiting the search to the last 5 years, from 1 May 2017 to 1 May 2022, with the aim to retrieve the most up-to-date information, since this is a rapidly evolving field of work. The following MeSH terms were used with the chosen Boolean connectors: “low level laser therapy” OR “photobiomodulation” AND “oral pathology” OR “oral lichen planus”. Duplicate scientific articles were first eliminated, and then a selection of the remaining articles was made based on title, abstract, and analysis of the full text considering the established inclusion/exclusion criteria. Doubts were resolved by reaching consensus between two operators (PLJ and PMA). The following inclusion criteria were considered: the language was English, Italian, Spanish and Portuguese and only patients over 18 years of age participating in non-randomized control trials (CTs) and randomized control trials (RCTs) were considered, thus excluding clinical cases, systematic reviews, meta-analyses and letters. We did not limit the sample size.

### 2.2. Data Extraction

Each paper was thoroughly analyzed considering the authors’ surnames and the year of publication, the research groups’ countries, the type of study carried out, the size of the sample, the type of PBM, the parameters used for the study, the treatments with which PBM was compared (with established doses and treatment protocols), the follow-up time, the scale or methods to analyze the effectiveness of the PBM, as well as the results obtained inn each study.

### 2.3. Quality Evaluation

The Newcastle–Ottawa Scale (NOS) was used in the collected studies to assess their methodological quality [27]. This scale is used to assign a score to each article. Articles with low quality scored between 0 and 3, those with moderate quality between 4 and 6, and those with high quality between 7 and 9.3.

An evaluation of the methodological quality of the selected articles was carried out using the NOS scale (Table 2). A series of parameters that the studies had to include (Selection, Comparability and Exposure) were analyzed in order to obtain the score to classify them based on information quality and the risk of bias. On average, the articles analyzed achieved a moderate score of 5.8 out of 9. Regarding the individual article scores, little heterogeneity was observed, since none of the studies presented low quality, five were of moderate quality, and two were of high quality. An aspect of quality that two studies did not meet was the selection of the control group that, in those cases, did not represent the community, because it came from groups at hospitals or dental clinics. Table 3 and Table 4 below show the most relevant data obtained from the different selected studies. These tables show the differences between the protocols followed by the different types of clinical trials that we analyzed.

## 3. Results

**Figure 1:**
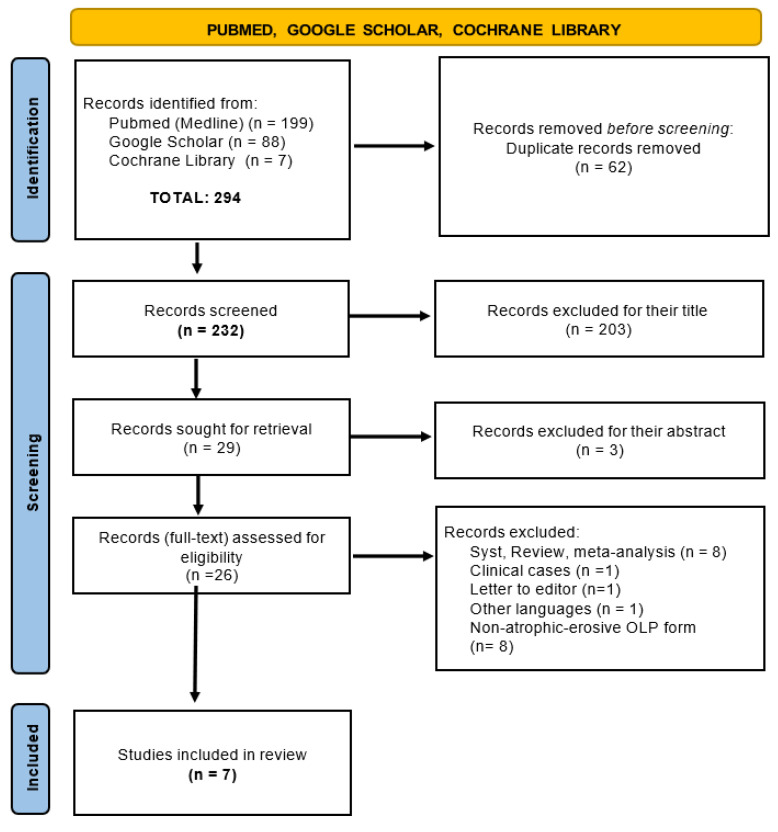
Flowchart.

A total of 294 articles from the databases were selected. After analyzing the title, abstract, full text and compliance with the inclusion/exclusion criteria, seven articles remained to be analyzed in this systematic review, as shown in Figure 1. All results were summarized and are synthesized in Table 3 and Table 4. The chosen articles were Gambino et al. (2018) [28], Mirza et al. 143 (2018) [29], Mutafchieva et al. (2018) [30], Lavaee et al. (2019) [31], Khater et al. (2020) [32], Nammour et al. (2021) [26] and Tarasenko et al. (2021) [33] as they met all the inclusion criteria proposed in this systematic review.

Of the seven selected articles, which were clinical trials, four were carried out in European countries, while the rest were carried out in the Middle East (Egypt, Iran and Saudi Arabia).

In relation to the sample size, there were high differences in the number of participants in our selection of studies. For example, Lavaee et al. [31] includes 8 patients, while Nammour et al. [26] included 96 patients, making it difficult to compare the results between these two studies.

Considering the location of the lesions, only in three studies (Gambino et al. [28], Lavaee et al. [31] and Nammour et al. [26]), the treatments focused on the buccal mucosa, in general, while in the other four studies, they were applied to lesions of the gums, palate, tongue and lips.

When analyzing the type of source used in PBM, as well as the parameters established in each study, we observed that in four cases the diode laser was used (Gambino et al. [28], Mirza et al. [29], Mutafchieva et al. [30] and Lavaee et al. [31]), while Khater et al. [32] and Tarasenko et al. [33] used the Nd–YAG laser at the same wavelength of 1064 nm, and Nammour et al. [26] used the He–Ne laser. The power used ranged between 25 mW in Lavaee et al. [31] and 3 W in Tarasenko et al. [33], while the minimum wavelength used was 630 nm, and the maximum one was 1064 nm.

Most studies analyzed lesions treated with topical corticosteroids as a control group, although some studies compared PBM with another type of a laser, e.g., (high-power laser) plus corticosteroids, as in Lavaee et al. [31], or with cold knife surgery, as in Tarasenko et al. [33]. The follow-up times of the studies were highly variable; therefore, it was not possible to establish any treatment pattern or extrapolate the best follow-up schedule to achieve the best results.

The scales or tests to evaluate and measure the effectiveness of the treatments were very diverse, although most studies used the VAS [29] (Visual Analog Scale), EI [29] (Efficiency Index) and Thongprasom tests [34]. This point is important, because there must be an objective tool that measures the effectiveness of a treatment.

Regarding the results, according to the study by Gambino et al. [28], corticosteroids were more effective in the short term, while PBM was better in the long term. In general, PBM worked perfectly by helping to reduce the signs and symptoms, such as pain, that are typical of atrophic–erosive LPO. Studies such as Mutafchieva et al. [30] concluded with a clinical improvement in 59.3% of the lesions, as did Khater et al. [32], with even a complete remission in 37.3% of the cases. Some authors such as Taresenko et al. [33] proposed using this therapy in combination with other treatments. They combined PBM or Low-Light Laser Therapy (LLLT) with High-Light Laser Therapy (HLLT), obtaining better 181 results for the remission of symptoms with HLLT.

## 4. Discussion

OLP is considered one of the potentially malignant disorders that can predispose to oral cancer, specifically, oral squamous cell carcinoma [34,35]. It is defined as a multi factorial process with different triggers that appears in various clinical types, among which is the atrophic–erosive form of the OLP, which is the most symptomatic and therefore requires a high number of treatments [6,9].

Conventional treatments used as first-line therapy are based on corticosteroids that are usually administered topically for their anti-inflammatory, immunomodulatory, antipruritic and vasoconstriction properties and are easy to apply (mouthwashes, ointments, creams, lotions and gels). However, they generate numerous side effects including psychological problems (mood swings) and physiological problems (e.g., fluid retention, thinning of the oral mucosa and systemic absorption) [15,35,36]. In contrast, PBM therapy, which applies an LLLT or LED to the cells of OLP lesions, is considered a safe treatment with no adverse effects and contributes to healing in the same way as corticosteroids [29,31]. 

The studies included in this systematic review showed that the biological effects of PBM treatment are beneficial compared to those of other conventional therapies such as corticosteroids [26,30,32] as shown by the reduction of symptoms such as pain and signs associated with LPO lesions, highlighted by de Carvalho et al. [24]. Other authors clarified that, in addition to PBM, there is another type of therapy, i.e., high-power laser (HLLT) [33] which, they claim, allow achieving significantly better results than PBM in the aforementioned parameters.

However, this contradicts the study by Gambino et al. [28], because it proposes PBM only as a long-term treatment instead of a short-term one.

Lavaee et al. [31] demonstrated in their clinical study that they did not find significant differences in efficacy between treatment with corticosteroids and PBM, which favored the latter.

In the case of atrophic–erosive OLP, all the anatomical areas of the oral cavity must be examined, whereas three of the selected articles focused only on one area (Nammour et al. [26], Gambino et al. [28] and Lavaee et al. [31]). A limited analysis can cause biases when evaluating some parameters and, according to Boñar-Álvarez et al. [37], all areas are important to properly evaluate the appearance of LPO.

In addition, it should always be kept in mind that PBM therapy is not applied to cure lesions but rather to improve the aforementioned clinical aspects, as reported in the studies by Mutafchieva et al. [30] and Khater et al. [32], who described that most of their patients obtained a moderate recovery, and some a complete one. These poor results are due to the lack of a long-term follow-up after therapy administration, as pointed out by Hanna et al. [21] and Carvalho et al. [24], which also makes it difficult to assess the possible side effects that PBM can produce in the long term, although we already know that it is much less invasive than other conventional treatments such as corticosteroids. In addition, it was observed in different studies such as Mirza et al. [29], Lavaee et al. [31] and Ferri et al. [38] that, in the short term, no side effect was been detected. Therefore, more studies are recommended on this aspect to determine with scientific evidence whether PBM could be applied as an alternative therapy to pharmacological treatments [18,26] or as a complement to photodynamic therapy [26] or HLLT [33].

This systematic review has limitations, such as the low number of patients and of randomized controlled clinical trials examined. In addition, a lack of follow-up was observed in most cases, as well as a high heterogeneity in the characteristics of the control groups. There was no consensus between the different methodologies used or the parameters analyzed to assess the effectiveness of PBM. For these reasons, we decided not to perform a meta-analysis. However, it stands out that all studies focused on the analysis of the same oral location and that the results obtained were similar, despite such a disparity of protocols. In general, the wide variety of information obtained from the included studies makes it difficult to establish a protocol based on precise values and parameters of PBM in relation to erosive–atrophic lesions of OLP. Therefore, it is still difficult to assess the influence of PBM on this disease [18].

## 5. Conclusions

It was shown that PBM as a treatment for atrophic–erosive OLP has provided favorable and very satisfactory results in relation to corticosteroids, which cause more complications; therefore, PBM could be used as an alternative or complementary therapy. There are no established protocols that determine the exact parameters to obtain the best results. The establishment of a standardized effective dose in future studies would allow a comparison of different protocols with greater reliability. In this sense, only a protocol establishing specific treatment parameters and evaluation measures would be of great clinical utility for professionals.

## Figures and Tables

**Table 1:** Components of the PICO strategy.

PICO Question	Characteristics
Patient	Patients with atrophic–erosive OLP
Intervention	PBM treatment with laser
Comparison	Drugs or laser off
Outcome	Remission of symptoms

**Table 2:** Quality evaluation: the Newcastle–Ottawa Scale [27].

Study	Selection	Comparability	Exposition	Note
Gambino et al., 2018 [28]	⋆⋆	⋆	⋆⋆	5
Mirza et al., 2018 [29]	⋆⋆	⋆⋆	⋆⋆	6
Mutafchieva et al., 2018 [30]	⋆⋆	⋆⋆	⋆	5
Lavaee et al., 2019 [31]	⋆⋆⋆	⋆⋆	⋆⋆	7
Khater et al., 2019 [32]	⋆⋆⋆	⋆⋆	⋆	6
Nammour et al., 2021 [26]	⋆⋆	⋆⋆	⋆	5
Tarasenko et al., 2021 [33]	⋆⋆	⋆⋆	⋆⋆⋆	7

⋆: Summary of the evaluation of risk of bias in the selected studies.

**Table 3:** Results.

Author, Year, Country	Type of Study	Sample Size (*n*)	Loc. of Lesions	PBM Type	Laser Parameters	Control Group	Follow- Up Type
Gambino et al., 2018 [28], Italy	Clinical trial	40	Oral mucosa	Diode laser gallium arsenide and aluminum (AlGaAs)	980 nm; 400 mW; 8 J/cm^2^; 10 s -point size 0.5 cm^2^; 8 sessions/1 week for 8 weeks	Twice daily propionate clobetasol to 0.05% gel with aqueous of hydroxyethyl-cellulos at 4% (100 g), in equal parts (50:50) for 8 weeks	After treatment (unspecified)
Mirza et al., 2018 [29] Saudi Arabia	Randomized controlled clinical trial	45	Oral mucosa, Tongue	Diode laser (unspecified)	630 nm; 10 mW/cm^2^ 1.5 J/cm^2^; 2.5 min; 1 cm^2^. 2 times per week, each 3 days. Max. 10 sessions	Topical; corticosteroids in mouthwash: Dexamethasone (0.5 mg in 5 mL of water for 5 min); 30 min later: Nystatin (30 drops during 5 min) 4 times per day for 1 month	Control group: weekly follow-up during intake. Once or twice a week and a year
Mutafchieva et al., 2018 [30] Bulgaria	Open clinical trial	12	Oral mucosa, Gum, Tongue, Labial mucosa, Palate	Diodelaser (unspecified)	810 nm; 0.5 W; 1.2 J/cm^2^; 30 s 3 times in a week	No control group	A month after treatment
Khater et al., 2019 [32], Egypt	Open clinical trial	24	Buccal mucosa, Gum, Tongue, Labial mucosa, Palate	Nd-YAG (neodymium) laser with Q shift	1064 nm; 0.5 W; 1.2 J/cm^2^; 30 s 3 times per week for 1 month	No control group	After treatment (unspecified)
Lavaee et al., 2019 [31], Iran	Double- blind ran- dom- ized clinical trial	8	Buccal mucosa	Diode laser InGaAlP (Indium Gallium Aluminum Phosphorus)	660 nm; 25 mW; 19.23 J/cm^2^	Stimulated laser + Topical corticosteroids (triamcinolone acetonide 0.10% 3 times in a day and 40 drops of 0.1% nystatin oral suspension for 4 min	3rd and 7th week of treatment
Nammour et al., 2021 [26], Belgium	Clinical trial	96	Buccal mucosa	Red light helium–neon (He–Ne) laser	635 nm; 0.1 W; 1415 J/ cm^2^; 40 s Every 48 hours for 6 weeks	Topical cortisone (0.05% clobetasol propionate gel); 3 times per day for 6 weeks	6 weeks, 1 month, 6 months and 12 months after treatment
Tarasenko et al. 2021 [33], Germany	Randomized controlled clinical trial with parallel arms and blinded examiner	75	Buccal mucosa, tongue, alveolar crest, palate, floor of mouth, lips	Nd–YAG (neodymium aluminum garnet laser)	1064 nm; 1.5 W or 3 W; 40 Hz; 15 s Postoperative 5.5 min	Scalpel with 5–10, size 5.0 microfilament sutures no painkillers	14, 30 days and 2 years after the operation

**Table 4:** Continued from previous page.

Author, Year	Intervention Type	Scales or Test to Measure Effectiveness	Results
Gambino et al., 2018 [28]	Therapy	OCT ^1^	The corticosteroid is more effective in the short term while PBM is better in the long-term.
Mirza et al. 2018 [29]	Therapy	EI ^2^ Thongprasom VAS ^3^	Control is significantly better at relieving pain, but PBM improved clinical signs. No side effects.
Mutafchieva et al., 2018 [30]	Therapy	Thongprasom VAS EI	General relief of symptoms, most with minor discomfort. Clinical improvement in 59.3% of the lesions. Moderate improvement in all cases, except for one that was cured.
Khater et al. 2019 [32]	Therapy	EI VAS Thongprasom	Clinical signs improved in 37.3% of the lesions with severe pain and discomfort presented by most patients before PBM; only mild discomfort remained. In almost all cases, there was a moderate recovery, which was complete in only one case.
Lavaee et al. 2019 [31]	Therapy	VAS EI Thongprasom SI ^4^	No statistically significant differences between intervention group and control group.
Nammour et al. 2021 [26]	Therapy	VAS REU ^5^	The treatments were beneficial in the absence of pain and recurrence but without significant differences between intervention group and con trol group.
Tarasenko et al., 2021 [33]	Therapy	VAS Pearson’s coeffi cient	Laser s more effective at the end of the first postoperative month. The com- bination LLLT + HLLT produced superior clinical performance compared to conventional surgical excision. However, the pain reduction was more signific-ant in HLLT than in LLLT.

^1^ OCT: Optical Coherence Tomography; ^2^ EI: Efficacy Index ^3^ VAS: Visual Analogic Scale; ^4^ SI: Clinical Severity Index; ^5^ REU: Reticular score = R; Erythematous score = E; and Ulcerative score = U).
